# HIV-1 Tat second exon limits the extent of Tat-mediated modulation of interferon-stimulated genes in antigen presenting cells

**DOI:** 10.1186/1742-4690-11-30

**Published:** 2014-04-17

**Authors:** Sami Kukkonen, Maria Del Pilar Martinez-Viedma, Nayoung Kim, Mariana Manrique, Anna Aldovini

**Affiliations:** 1 Department of Pediatrics, Harvard Medical School, Department of Medicine, Boston Children’s Hospital, 300 Longwood Avenue, Boston, MA 02115, USA

**Keywords:** Immature dendritic cells (iDC), Monocyte-derived macrophages (MDM), IFN-stimulated genes (ISG), Tat, Human immunodeficiency virus type 1 (HIV-1), Antigen presenting cells (APC)

## Abstract

**Background:**

We have shown that HIV-1 Tat interaction with MAP2K3, MAP2K6, and IRF7 promoters is key to IFN-stimulated genes (ISG) activation in immature dendritic cells and macrophages.

**Results:**

We evaluated how Tat alleles and mutants differ in cellular gene modulation of immature dendritic cells and monocyte-derived macrophages and what similarities this modulation has with that induced by interferons. The tested alleles and mutants modulated to different degrees ISG, without concomitant induction of interferons. The first exon Tat_SF2_1-72 and the minimal transactivator Tat_SF2_1-58, all modulated genes to a significantly greater extent than full-length wild type, two-exon Tat, indicating that Tat second exon is critical in reducing the innate response triggered by HIV-1 in these cells. Mutants with reduced LTR transactivation had a substantially reduced effect on host gene expression modulation than wild type Tat_SF2_. However, the more potent LTR transactivator Tat_SF2_A58T modulated ISG expression to a lower degree compared to Tat_SF2_. A cellular gene modulation similar to that induced by Tat and Tat mutants in immature dendritic cells could be observed in monocyte-derived macrophages, with the most significant pathways affected by Tat being the same in both cell types. Tat expression in cells deleted of the type I IFN locus or receptor resulted in a gene modulation pattern similar to that induced in primary immature dendritic cells and monocyte-derived macrophages, excluding the involvement of type I IFNs in Tat-mediated gene modulation. ISG activation depends on Tat interaction with MAP2K3, MAP2K6, and IRF7 promoters and a single exon Tat protein more strongly modulated the luciferase activity mediated by MAP2K3, MAP2K6, and IRF7 promoter sequences located 5′ of the RNA start site than the wild type two-exon Tat, while a cysteine and lysine Tat mutants, reduced in LTR transactivation, had negligible effects on these promoters. Chemical inhibition of CDK9 or Sp1 decreased Tat activation of MAP2K3-, MAP2K6-, and IRF7-mediated luciferase transcription.

**Conclusions:**

Taken together, these data indicate that the second exon of Tat is critical to the containment of the innate response stimulated by Tat in antigen presenting cells and support a role for Tat in stimulating cellular transcription via its interaction with transcription factors present at promoters.

## Background

Tat is among the first genes expressed during HIV-1 infection and functions as a transcription elongation factor for viral gene expression [1-4]. It is also expressed before integration of the infecting viral genome [5]. Most of the evidence on Tat function has been obtained in experiments carried out with an 86 amino acid Tat protein derived from the laboratory-adapted HIV-1 strains HXB2 or NL4-3, although in most primary strains Tat is a 101 amino acid protein. The first exon of 72 amino acids is sufficient for transactivation of the HIV LTR and contains a basic domain and a cysteine-rich domain, whose cysteines are critical to protein function [6-8]. Deletion of the second exon, which can vary in size, does not substantially affect HIV-1 LTR transactivation in transfection assays but leads to reduce viral replication and activation of NF-kB [9]. Two domains in this exon, (RGD and ESKKKVE), are highly conserved between human and other primate lentiviruses, but their significance is not fully understood. Furthermore, findings from HIV-2 and SIV Tat suggest that this exon also contributes to optimal transactivation and to chronic SIV replication in vivo [10-12].

Tat increases HIV-1 gene expression by functioning as an elongation factor and interacting with TAR, a RNA sequence present at the beginning of the HIV viral transcripts, and with the host cell factors CDK9 and cyclin T1, which promotes auto-phosphorylation of the C-terminus of CDK9 [3,4,13-18]. CDK9 is the catalytic subunit of P-TEFb, which phosphorylates the C-terminal domain of the large subunit of RNA polymerase II, which in turn affects transcript elongation [3]. Tat is also thought to interact with additional transcriptional regulators, including the protein kinase PKR, Sp1 and the transcriptional coactivators p300 and the CREB-binding protein (CBP) [4,19-21]. The interaction of Tat with these key host cell transcriptional regulators might be expected to also affect host cell gene expression. Indeed, there is considerable evidence that Tat can affect the physiology of T lymphocytes, neurons and antigen presenting cells [22-26]. For example, exposure of peripheral blood mononuclear cells to exogenous Tat, which can bind and enter uninfected cells, affects proliferative responses after exposure to recall antigens [25,26]. The evidence that Tat can influence host cell behavior makes it important to determine precisely how Tat affects the gene expression program of its various host cells.

We explored the effects of HIV-1 and its Tat transactivator on myeloid immature dendritic cells (iDC) and found that HIV-1 infection or Tat production can induce expression of ISG [23]. Several of the genes induced by HIV-1 and its Tat transactivator encode chemokines that recruit activated T-cells and macrophages, the ultimate target cells for the virus. HIV-1 Tat can reprogram host dendritic cell gene expression to facilitate expansion of the infection and by itself can recapitulate the virus-induced expression of ISG [23]. Activation of ISG is mediated by Tat interaction with the promoters of two kinases, MAP2K3 and MAP2K6, and of IRF7 [27]. Sequences upstream of the RNA start site are sufficient for the Tat-mediated increased transcription of these genes. The consequence of these interactions is the activation of p38MAPK- and IRF7-regulated pathways [27]. A Tat-mediated species-specific increase in ISG was observed in human and Rhesus macaque iDC and monocyte-derived macrophages (MDM) but not in the same cells from Sooty Mangabey (SM) and African Green Monkeys (AGM), in which SIV establishes a persistent non pathogenic infection [28]. Our results link the differential induction of ISG to species-specific differences in disease susceptibility. We also found that in HIV-infected primary CD4+ T-cells apoptosis is triggered by the Tat-dependent activation of PTEN-FOXO3a-Egr1 and p53 pathways, which converge on the FOXO3a transcriptional activator and that this activation provides a mechanism for HIV-1-associated CD4+ T cell death [23,29]. Therefore Tat can affect different cellular pathways in different cell types, suggesting that interaction with cellular proteins differentially available or cell-dependent chromatin accessibility may be critical to the observed gene modulation.

To investigate which domains of Tat are critical to the host-pathogen interactions that are Tat-dependent during HIV infection, we evaluated a variety of Tat-mutants and found that in antigen presenting cells (APCs) as iDC and MDM, the second exon of Tat reduces innate immune responses that are maximal when a single exon Tat is expressed. This suppression could be critical to modulate virus production in these cells. Furthermore the analysis of Tat mutants supports a mechanism of Tat-transactivation of cellular genes similar, but not identical, to that described to increase HIV LTR-driven gene expression.

## Results

Our previous finding indicated that HIV Tat can affect the gene expression of iDC and induce expression of some of the ISG [23] via interaction with MAP2K3 and MAP2K6, and with IRF7 [27]. Promoter sequences located 5′ of the RNA start site are sufficient to observe an increase transcription of a reporter gene but Tat-mediated modulation is unlikely due to direct DNA binding and more likely dependent on the interaction with transcription factors [27]. In order to characterize which domains of the Tat protein are critical to cellular gene modulation we used a combination of genetic and biochemical approaches aimed at mapping the role of different Tat domain in cellular gene modulation.

### Tat mutants lacking the second exon modulate cellular gene expression more significantly than wild type Tat

**Figure 1:**
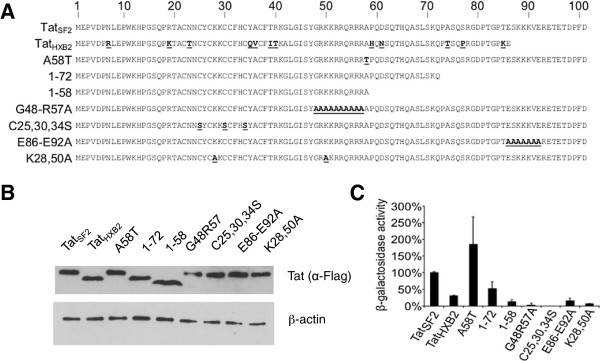
**Recombinant adenoviruses were generated expressing three alleles and six mutants of Tat. (A)** Sequences of the Tat alleles and Tat_SF2_ mutants. Amino acids that differ from the wild-type Tat_SF2_ are in bold and underlined. **(B)** Western Blot analysis of Tat alleles and mutant in iDC. **(C)** The three Tat alleles and six Tat mutants were expressed in HeLa cells that have the β-galactosidase gene under the control of the HIV-1 LTR promoter. Transactivation activity was measured by a β-galactosidase assay and compared to the activity of the wild-type Tat_SF2_.

Tat alleles and mutants with different phenotypes have been described extensively [27,30-36]. We reasoned that testing these known alleles and mutants in modulation of cellular transcription would permit a comparison with their effect on viral transcription from the HIV LTR. Three Tat alleles and six mutants were cloned into an adenovirus vector and the derived viruses were tested for Tat protein expression and transactivation activity of the Tat protein they express (Figure 1). The Tat gene present in these adenoviruses is under the control of a tet promoter and the expression of Tat mRNA requires the expression of the tet transactivator (tTA) in cells. One of the Tat proteins used in these experiments was from HIV-1_HXB2_, which is a clone of HIV-1_LAI_ that was adapted to cell-culture [37,38]. Because of a premature termination codon in the second exon, Tat_HXB2_ has 86 amino acids instead of the 101 typically found in Tat of HIV-1 of patients, for instance Tat_SF2_ (Figure 1A). The Tat_HXB2_ was less efficient in LTR transactivation than Tat_SF2_ (Figure 1C). Comparison of these Tat alleles should reveal how the modulation induced by the Tat protein from a cell culture-adapted virus differs from that of a Tat protein more common in patients. HIV-1_SF13_ replicates faster than HIV-1_SF2_ and Tat_SF13_ is a more potent transactivator than Tat_SF2_[30]. The amino acid causing the difference between the two was mapped to position 58 (Figure 1A) [30]. The A58T mutation in Tat_SF2_A58T recapitulates the transactivation properties of Tat_SF13_ (Figure 1C). If Tat transactivates host genes via the same mechanism used for the transactivation of HIV-1 genes under the LTR promoter Tat_SF2_A58T should differ from Tat_SF2_ in its effect on host gene expression.

We generated two Tat mutants lacking the second exon: Tat_SF2_1-72 and Tat_SF2_1-58 (Figure 1A). Tat_SF2_1-72 has all the amino acids encoded by the first exon. Tat_SF2_1-58 is the minimal transactivator [31,32]. Tat mutants that have less than the 58 amino-terminal residues are no longer able to transactivate genes under the HIV-1 LTR promoter. Comparing the effect of these mutants to wild-type Tat_SF2_ should reveal what role the second exon plays in modulation of host gene expression. Tat_SF2_1-72 caused lower LTR transactivation than wild type Tat_SF2_ and transactivation was also present at 19% of the amount observed with Tat_SF2_ with the minimal transactivator Tat_SF2_1-58 (Figure 1C). Tat_SF2_C25,30,34S (Figure 1A) did not transactivate the lacZ gene controlled by the HIV-1 LTR promoter (Figure 1C). Lysine residues at positions 28 and 50 are acetylated by the histone acetyl transferases (HAT) p300 and p300/CBP-associating factor (PCAF) and the latter also interacts with hGCN5 [33-35]. The Ad-Tat_SF2_K28,50A mutant (Figure 1A) was used to investigate whether histone acetylation plays in regulation of the host transcriptome. These mutations lowered LTR transactivation (Figure 1C). Comparing these mutants to the wild type should tell us whether host gene transcription is transactivated by a similar mechanism as HIV-1 genes. HIV and SIV Tat proteins have a highly conserved amino acid motif, ESKKKVE in their C-terminus. In the mutant Tat_SF2_E86-E92A this region was substituted with alanines (Figure 1A) and was used to find out whether this conserved motif is important in modulation of the host transcriptome. These mutations lowered the LTR transactivation activity to a level similar to the minimal transactivator (Figure 1C). We also utilized the mutant Tat_SF2_G48-R57A (Ad-Tat_SF2_G48-R57A), in which the nuclear localization signal residues 48 to 57 were substituted by alanines [27,36]. This mutant does not enter the nucleus and therefore cannot affect cellular transcription, providing a significant negative control in gene expression experiments.

We evaluated the steady state accumulation of Tat alleles and mutants in iDC by Western Blot and studied the subcellular localization of the FLAG-tagged versions of these mutants by immunofluorescence microscopy. All Tat proteins accumulated approximately at similar levels (Figure 1B) and localized in the nucleus with the exception of mutant Tat_SF2_G48-R57A (not shown).

In our previous experiments Ad-Tat_HXB2_ caused the induction of ISG in iDC [23,28]. To investigate how different Tat alleles and Tat mutants differ in their effect on host gene expression, human monocytes were differentiated into iDC, and then infected with the Tat-expressing adenoviruses and Ad-tTA alone as a control. Cellular gene expression was investigated in RNA isolated 5, 10, and 20 h post-infection with the Affymetrix Human Genome U133 Plus 2.0 Array. The results for Tat alleles and mutants were compared to HIV-1 infection of iDC collected 10 and 14 days post infection and previously analyzed with the Affymetrix Human Genome U133 Array (HG 133). In previous experiments Ad-Tat caused the up-regulation of ISG [23]. Therefore, to compare the mimicry of interferons caused by Tat to the effects caused by Type I and II interferons, we also treated iDC with interferon α, β, and γ.

**Figure 2:**
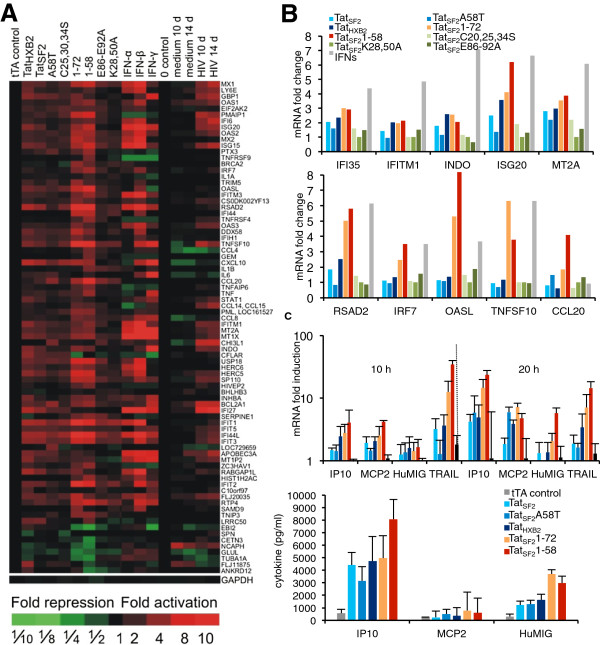
**ISG modulation by Tat alleles and mutants in iDC. A**. RNA expression analysis in iDCs expressing Tat alleles and mutants. Gene expression fold induction is shown for RNA isolated 10 h after adenovirus infection or interferon treatment, and 10 and 14 days after HIV-1 infection. Data for HIV-1 infection, previously published [23], are included here for direct comparison. GAPDH levels were used to normalize the results of the individual samples. **B**. Microarray mRNA levels of selected ISG in iDC expressing Tat alleles and mutants or exposed to IFNs (10 h time point). RNA fold change is reported as the ratio of the signal in the experimental sample compared to the control. **C**. Gene expression analysis of selected ISG RNA levels by RT-PCR (upper panel) and protein levels by ELISA (lower panel, 20 h post-infection) in cell lysates and their respective supernatants of iDC from five donors. RNA fold change compared to that of control cells infected with Ad-tTA, was evaluated using the threshold cycle (Ct) number normalized for β-actin Ct.

We focused on transcripts whose expression levels changed at least two-fold at two consecutive time points or 3-fold or more at one time point in any of the Tat alleles or mutants compared to those modulated by the control Ad-tTA. Expression of Tat_SF2_ caused the up-regulation of sixty-seven genes and the down-regulation of nine genes according to these criteria (Figure 2). When an Ingenuity Pathway Association analysis (IPA) of this group of genes was carried out we found significant association with the canonical pathway and the biological functions listed in Table 1. The various Tat alleles and mutants modulated similar sets of host genes but the extent of gene modulation differed (Table 2). Tat_SF2_1-72 and Tat_SF2_1-58 caused more significant gene modulation than the wild type Tat alleles and mutants that retain the second exon. This was true when the data were analyzed in a number of ways: a larger number of genes were affected (Table 2), the changes in levels of transcription were of larger magnitude (Figure 2A, B), and the significance of modulation of different pathways was higher (Table 2, data reported for gene function annotation associated with mechanism of infection). The Tat_HXB2_ allele, which has a shortened second exon encoding 14 amino acids instead of the 29 in Tat_SF2_, also caused a more extensive gene modulation than the wild-type Tat_SF2_. This indicates that the amino acids encoded by the second exon of Tat diminish the impact Tat has on host gene expression. Tat_SF2_A58T, which recapitulates the phenotype of Tat_SF13_, modulated similar sets of genes but to a lower degree than Tat_SF2_. Tat_SF2_A58T is a more potent transactivator of genes expressed under the control of the HIV-1 LTR promoter than Tat_SF2_[30]. The mutant Tat_SF2_K28,50A and Tat_SF2_C25,30,34S, which are both inactive in LTR transactivation, caused significantly less gene modulation of the host transcriptome than the wild-type Tat_SF2_ (Figure 2 and Table 2). The mutations in the conserved region in the second exon encoding, introduced in the Ad-Tat_SF2_E86-E92A mutant, also reduce substantially the effect of Tat on host gene expression (Figure 2 and Table 2). As this domain is deleted in Tat_HXB2_ and deletion of entire second exon actually increases Tat-mediated gene modulation, indicating that this entire exon is not necessary for this function, it is possible that these residues play a role in the correct folding of Tat and the introduced mutations disrupt its structure too drastically to be compatible with function. Therefore this mutant does not provide any information in the role played by this domain of Tat in gene modulation.

**Table 1:** Ingenuity systems pathway analysis of genes found modulated by HIV and Tat in primary iDC and MDM

**Ingenuity canonical pathways**	**p-value**		
	**iDC**	**MDM**	**Common to both**
Interferon signaling	7.59E-09	9.94E-10	2.54E-11
Pathogenesis of multiple sclerosis	4.44E-08	1.45E-07	1.05E-09
Role of pattern recognition receptors in recognition of bacteria and viruses	1.24E-06	3.95E-07	2.51E-07
Activation of IRF by cytosolic pattern recognition receptors	9.36E-06	3.87E-05	4.93E-06
Ingenuity biological functions			
Antimicrobial response	8.44E-12	2.68E-10	2.82E-11
Cell-mediated immune response	2.35E-10	7.57E-08	1.23E-10
Immune cell trafficking	2.35E-10	7.57E-08	1.23E-10
Infection mechanism	4.35E-08	1.45E-07	1.05E-09
Antigen presentation	4.10E-09	7.72E-07	2.09E-08

**Table 2:** Ingenuity systems pathway analysis of genes found up-regulated by Tat and mutants iDC

	**# Upregulated**	**# Downregulated**	**Infection mechanism (p-value)**
Ad-Tat_SF2_	67	9	3.30E-06
Ad-Tat_SF2_A58T (SF13)	49	12	5.63E-05
Ad-Tat_HXB2_	113	15	2.20E-12
Ad-Tat_SF2_1-72	205	31	1.57E-14
Ad-Tat_SF2_1-58	291	121	6.51E-16
Ad-Tat_SF2_C20,25,34S	41	7	1.28E-02
Ad-Tat_SF2_K28,50A	26	7	1.37E+00
Ad-Tat_SF2_E86-92A	47	19	1.18E+01
IFN-α 100 U/ml	103	9	
IFN-β 100 U/ml	248	49	
IFN-γ 100 U/m	347	126	
Type I IFNs			4.69E-10

Many but not all of the genes up-regulated by the HIV Tat_SF2_ could also be found modulated by interferons. However the patterns observed when cells were exposed to Type I and Type II IFN were different, and consisted of a larger number of genes modulated to a higher degree. The comparative analysis revealed that Tat_SF2_1-72 modulation pattern correlated more closely is with IFN-β (R^2^ = 0.6630) than IFN-α (R^2^ = 0.4580) or IFN-γ (R^2^ = 0.2948) and that type I IFNs are more similar to each another in their impact on gene modulation (IFN-α vs. IFN-β R^2^ = 0.8833; IFN-α vs. IFN-γ R^2^ = 0.2290; IFN-β vs. IFN-γ R^2^ = 0.3158) than to Tat. However, Tat and Type I IFN patterns are dissimilar enough to make it unlikely that the effects observed in presence of Tat are due to stimulation of IFN production. Indeed, no expression of Interferon α, β, or γ genes could be detected by RT-qPCR and the corresponding proteins were not detected in the supernatant of Tat_SF2_ expressing iDC (not shown).

The effect that Tat alleles and mutants had on host gene expression was analyzed also by RT-qPCR in iDC. RNA was isolated at multiple time points post-infection and the following RNAs were quantified: glyceraldehyde-3-phosphate dehydrogenase (GAPDH), as a control gene used for normalization, Tat, to correct for possible differences in infectivity, and a subset of ISG that includes IP-10, TRAIL, MCP-2, and HuMig that were observed modulated in the microarray expression analysis. The mutants lacking the second exon, Tat_SF2_1-72 and Tat_SF2_1-58, caused a more pronounced up-regulation of the four-cytokine mRNAs 20 h post-infection than Tat_SF2_ (Figure 2C, top). We next evaluated changes at the protein level of some ISG by measuring by ELISA the amount of IP-10, MCP-2, and HuMig in the growth medium. The ELISA results were similar to the RT-qPCR, confirming that the highest expression levels were found with the mutants lacking the second exon, Tat_SF2_1-72 and Tat_SF2_1-58 (Figure 2C, bottom). We concluded that a single exon- or a shorter Tat, down to a size still competent of LTR transactivation (58 aa-Tat), has the greatest effect on cellular gene modulation while the presence of the second exon of Tat substantially reduces cellular gene modulation, in particular the induction of cytokines that are part of the innate response triggered in iDC by viruses.

### Comparative analysis of Tat-mediated gene expression modulation in iDC and MDM

**Figure 3:**
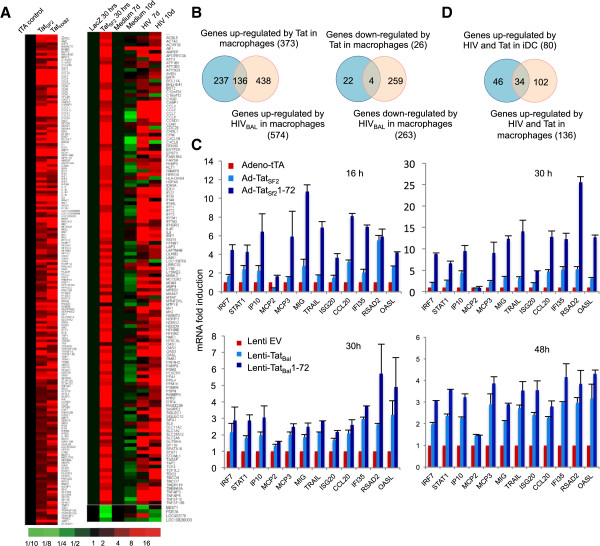
**Comparative analysis of Tat-mediated gene modulation in iDC and MDM. A**. Microarray RNA levels in MDM infected with Ad-Tat_SF2_ or Ad-Tat_HXB2_ (left panel) and in MDM infected with Ad-Tat_HXB2_ for 30 h or with HIV_Bal_ for 7 and 10 days (right panel). **B**. Venn diagrams representing total number of genes modulated by Tat or HIV-1 in MDM and the overlapping subset similarly modulated in the two conditions. **C**. Modulation of a subset of ISG in MDM evaluated by quantitative RT-PCR. Tat_SF2_ and Tat_SF2_72 were expressed using an adenoviral vector and RNA was purified 16 and 30 h after infection. Tat_Bal_, and Tat_Bal_1-72 were expressed using a lentiviral vector and RNA was purified 30 and 48 h after infection from cells sorted 24 h after infection using a GFP maker present in the lentivector. Results are reported as the average (*n*-fold) induction of three independent experiments relative to that of the control sample infected with the background vector. Values are normalized using 18S RNA and Tat levels. Significant differences between the cumulative levels of all the evaluated ISG expressed in Tat_SF2_ and in Tat_SF2_1-72 were detected at 16 and 30 h (p < 0.0001 for both time points, *T*-test) and also between Tat_Bal_, and Tat_Bal_1-72 at 30 (p = 0.0004) and 48 h (p < 0.0001). Significant differences could also be detected when individual gene expression was compared. P values for gene fold inductions in Tat_SF2_1-72 samples compared to Tat_SF2_ were: IRF7-16 h: p = 0.03, IRF7-30 h: p = 0.006; STAT1-16 h: p = 0.04, STAT1-30 h: p = 0.03; IP10-30 h: p = 0.04; MIG-16 h: p = 0.0001, MIG-30 h: p = 0.03; TRAIL-16 h: p = 0.04; ISG20-16 h: p = 0.02, ISG20-30 h: p = 0.002; CCL20-16 h: p = 0.003, CCL20-30 h: p = 0.03; IFI35-16 h and 30 h: p = 0.03; RSAD2-30 h: p = 0.01; OASL-16 h: p = 0.01, OASL-30 h: p = 0.008. P values for gene fold inductions in the Tat_Bal_ samples compared to the Tat_Bal_1-72 were: MCP3-30 h: p = 0.008; TRAIL-30 h: p = 0.002; ISG20-30 h: p = 0.01; IFI35-30 h: p = 0.05, IRF7-48 h: p = 0.02; STAT1-48 h: p = 0.02; MIG-48 h: p = 0.02. **D**. Number of genes modulated by Tat and HIV-1 in MDM and in iDC and subset similarly modulated in the two cell types.

Previous observations indicated that HIV infection of MDM also induces a significant number of ISG and that this effect can be attributed to Tat [39-42]. To extend our observation to antigen presenting cells that derive from the same lineage as iDC we evaluated the modulation of gene expression using HIV-1 infection and adenoviruses expressing Tat_SF2_ and Tat_HXB2_ in macrophages derived from the monocytic cell line THP-1 (THP-Mac) and in primary MDM, in which we confirmed those observations (Figure 3A). We also focused on the genes similarly regulated by HIV and Tat_HXB2_ that were differentially regulated when compared to the control and were at least two-fold upregulated or downregulated. 136 genes fit this definition (Figure 3A, B). When a subset of genes was investigated in three independent donors, we confirmed that although there is donor variability, the induction of a subset of ISG is detected across different donors (not shown). The IPA analysis for these genes is reported in Table 1. To extend these observations to a Tat protein from a different strain, HIV-1_Bal_ and evaluate the effect of one vs. two exon Tat in MDM, we also investigated how Tat_Sf2_ and Tat_Bal_ and their corresponding single-exon, 72 amino acid Tat proteins modulate expression of a subset of ISG by quantitative RT-PCR. We found that Tat_Bal_ was similar to Tat_SF2_ in its cellular gene modulation and that, as observed in iDC, the single-exon Tat protein modulated most of these genes to a significantly higher degree. When the cumulative levels of gene expression of all the ISG genes stimulated by the one-exon Tat were compared to that stimulated by the wild type Tat the difference of this modulation was statistically significant for both Tat alleles (p < 0.0001 for Tat_SF2_ and Tat_SF2_1-72 and p < 0.0004 for Tat_Bal_ and Tat_Bal_1-72) (Figure 3C).

Although not identical, there were significant similarities between the sets of genes modulated by HIV and Tat in iDC and in MDM (Figure 3D). The subset of genes similarly regulated in iDC and MDM are listed in Table 3. Their association with specific cellular pathways, including the statistical significance of the association, is also reported (Table 1, last column). The most significantly affected pathways were interferon signaling, activation of IRF by cytosolic pattern recognition receptors (PRR), and role of PRR in recognition of bacteria and viruses. This comparative analysis indicates that Tat can modulate the innate response that pathogens trigger in both types of APC and that utilizes the second exon to contain the extent of such response.

**Table 3:** Lists of genes similarly modulated by HIV and Tat in iDC and MDM

**Gene symbol**	**Entrez gene name**	**Gene bank**
AIF1	Allograft inflammatory factor 1	NM_004847
ATF3	Activating transcription factor 3	AB066566
BST2	Bone marrow stromal cell antigen 2	NM_004335
CCL3	Chemokine (C-C motif) ligand 3	NM_002983
CCL5	Chemokine (C-C motif) ligand 5	AF043341
CCL7	Chemokine (C-C motif) ligand 7	NM_006273
CCL8	Chemokine (C-C motif) ligand 8	AI984980
CDC20	Cell division cycle 20 homolog (S. cerevisiae)	NM_004295
CXCL10	Chemokine (C-X-C motif) ligand 10	NM_001565
CXCL9	Chemokine (C-X-C motif) ligand 9	NM_002416
HSPA6	Heat shock 70kDa protein 6 (HSP70B')	NM_002155
IDO1	Indoleamine 2,3-dioxygenase 1	M34455
IFI27	Interferon, alpha-inducible protein 27	NM_005532
IFI35	Interferon-induced protein 35	BC001356
IFI44	Interferon-induced protein 44	NM_006417
IFIT2	Interferon-induced protein with tetratricopeptide repeats 2	AA131041
IFIT3	Interferon-induced protein with tetratricopeptide repeats 3	AI075407
IRF7	Interferon regulatory factor 7	NM_004030
ISG15	ISG15 ubiquitin-like modifier	NM_005101
KPNB1	Karyopherin (importin) beta 1	AC004941
LY6E	Lymphocyte antigen 6 complex, locus E	NM_002346
MX1	Myxovirus (influenza virus) resistance 1, interferon-inducible protein p78 (mouse)	NM_002462
MX2	Myxovirus (influenza virus) resistance 2 (mouse)	NM_002463
OAS1	2',5'-oligoadenylate synthetase 1, 40/46kDa	NM_001255
OAS2	2'-5'-oligoadenylate synthetase 2, 69/71kDa	NM_002535
OASL	2'-5'-oligoadenylate synthetase-like	NM_003733
SLC11A2	Solute carrier family 11 (proton-coupled divalent metal ion transporters), member	2AF046997
SLC1A2	Solute carrier family 1 (glial high affinity glutamate transporter), member 2	NM_004171
STAT1	Signal transducer and activator of transcription 1, 91kDa	BC002704
TNFAIP3	Tumor necrosis factor, alpha-induced protein 3	AI738896
TNFAIP6	Tumor necrosis factor, alpha-induced protein 6	NM_007115
TNFSF10	Tumor necrosis factor (ligand) superfamily, member 10	AW474434
TRIM22	Tripartite motif containing 22	AA083478
USP18	Ubiquitin specific peptidase 18	NM_017414

### Tat-mediated modulation of ISG does not require Type I IFN production

**Figure 4:**
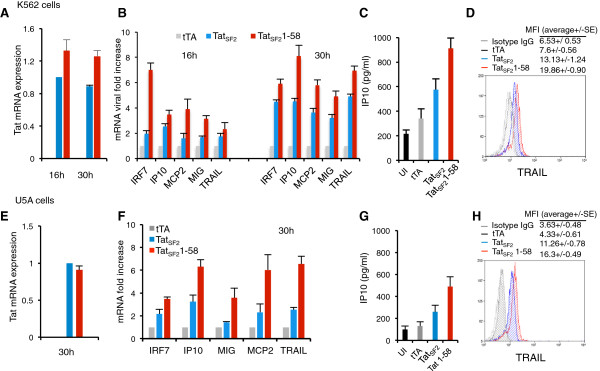
**Induction of ISG by HIV Tat**_**SF2 **_**in K562 and U5A cells (4E-4H) cells.** Detection of Tat RNA expression by RT-qPCR in K562 and U5A cells 16 and 30 h **(A)** and 30 h **(E)** after adenoviral infection. **B**. Detection of ISG RNA expression by RT-qPCR after adenoviral infection in K562-DC at 16 and 30 h. Statistical significance was detected by *T* Test when comparing Tat_SF2_ and Tat_SF2_1-58 values at 16 h for IRF-7 (p = 0.02), and for the ISG RNA values grouped together (p = 0.0007); at 30 h for IP-10 (p = 0.04), MCP2 (p = 0.05), MIG (p = 0.049), TRAIL (p = 0.01), and for the ISG RNA values grouped together (p = 2.19E-06). **C**. IP10 ELISA of Tat_SF2_ and Tat_SF2_1-58 expressing K562 supernatants; p = 0.0002 by ANOVA and Bonferroni’s multiple comparison test was significant for all pairs except UI and tTA; significant differences were also found by *T*-Test between, tTA and Tat_SF2_ (p = 0.05), tTA and Tat_SF2_1-58 (p = 0.0001) and Tat_SF2_ and Tat_SF2_1-58 (p = 0.047). **D**. Protein analysis of TRAIL levels by ICS in K562: tTA and Tat_SF2_ (p = 0.043, *T*-Test) and Tat_SF2_ and Tat_SF2_1-58 (p = 0.041). **F**. Detection of ISG RNA expression by RT-qPCR after adenoviral infection in U5A cells at 30 h. Statistical significance was detected when comparing Tat_SF2_ and Tat_SF2_1-58 values for IRF-7 (p = 0.03,*T*-Test), IP-10 (p = 0.01), TRAIL (p = 0.02), and for the cumulative values of all the ISG (p = 0.0046). **G**. IP10 ELISA of Tat_SF2_ and Tat_SF2_1-58 of infected U5A supernatants; p = 0.0008 by 1-way ANOVA, Bonferroni’s multiple comparison test was significant for all pairs except UI and tTA; significant differences were also found by *T*-Test between tTA and Tat_SF2_1-58, (p = 0.003), tTA and Tat_SF2_ (p = 0.008) and Tat_SF2 and_ Tat_SF2_1-58 (p = 0.01). **H**. Protein analysis of TRAIL levels by ICS in K562: tTA and Tat_SF2_ (p = 0.03, *T*-Test) and Tat_SF2 and_ Tat_SF2_1-58 (p = 0.04, *T*-test). RNA fold induction represents the ratios between the RT-PCR result obtained for cells expressing Tat or Tat1-58 and that obtained for Ad-tTA infected cells. Ct values in all samples were normalized using β-actin expression levels. Tat amount in the 30 h wild-type Tat_SF2_ infected sample was assigned an arbitrary value of 1.

In iDC half of the 291 genes that were up-regulated by Tat_SF2_1-58 were also up-regulated by interferon (IFN)-α or IFN-β. Furthermore, half of the 121 down-regulated genes by IFN-α or IFN-β were also similarly affected by Tat (Figure 2A). It is reasonable to assume that the induction of ISG upon HIV infection or Tat expression could results from the induction of IFN. As mentioned above, interferons could not be detected in the supernatants of iDC or MDM cultures by ELISA or at the RNA level by RT-qPCR. However it is possible that amounts below levels of assay limit of detection were produced. To test whether gene modulation could occur in the absence of type I interferons, we evaluated the effect of Tat expression in two cell lines that can be differentiated into iDC, K562 and U5A. The first is a human erythromyeloblastoid leukemia cell line derived from a chronic myeloid leukemia patient in blast crisis in which the entire Type I IFN locus is deleted [43]. In U5A cells the type I IFN receptor chain 2 is deleted [44,45]. In iDC derived from these lines the induction of a subset of ISG could be detected both at RNA and protein levels after Tat expression (Figure 4). The expression of Tat_SF2_ in K562 cells, monitored by RT-qPCR, resulted in increased mRNA levels of all the genes we tested. (Figure 4A, B). The transcription factor IRF7 was up-regulated in these cells as it was in primary iDC infected with Tat_SF2_. We also observed up-regulation of IP-10, measured by ELISA, and TRAIL, measured by intracellular staining (ICS) and reported as median fluorescence intensity (MFI) (Figure 4C and D). As in primary cells, the expression was significantly higher when the Tat_SF2_1-58 was expressed. When modulation of cellular genes by Tat expression was evaluated in U5A cells, which are unresponsive to Type I IFNs, the results were similar to those observed in K562 and confirmed the IFN-independent activation of ISG (Figure 4E-H). These results indicate that HIV Tat expression results in modulation of ISG and that this induction is not the result of production of IFN-α or IFN-β. Therefore we can conclude that Tat can modulate an innate immune response similar to that stimulated by Type I IFNs in the absence of any IFN.

### Tat modulation of ISG depends on interactions with cellular transcription factors

**Figure 5:**
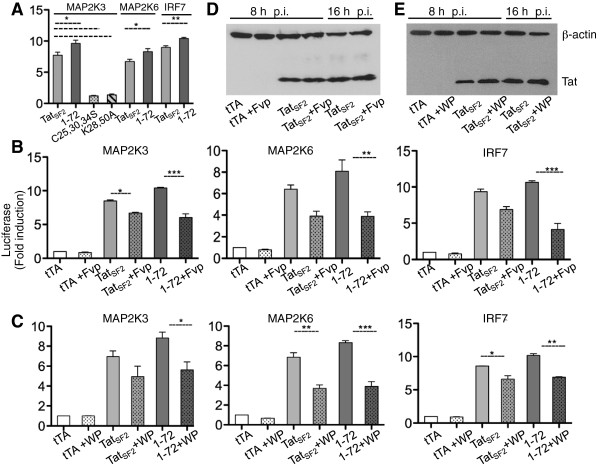
**Tat modulation of ISG requires P-TEFb and Sp1. A**. Luciferase activity in THP-Mac lysates expressing the wild-type Tat_SF2_, the single exon Tat_SF2_1-72, Tat_SF2_C25,30,34S or Tat_SF2_K28,50A and transfected with IRF7-, MAP2K6-, or MAP2K3-luciferase reporter vectors (the promoter used in the transfected vector is indicated above the bars). Significantly different levels of luciferase activity could be detected between Tat_SF2_ and Tat_SF2_1-72 for all three promoters (MAP2K3: p = 0.04; MAP2K6: p = 0.03; IRF7: p = 0.004, *T* Test). **B**. Luciferase activity in lysates from THP-Mac expressing the wild-type Tat_SF2_, the single exon Tat_SF2_1-72 and transfected with MAP2K3-, MAP2K6-, or IRF7-luciferase reporter vectors. “ + Fvp” indicates treatment with Flavopiridol (70 nM). **C**. Luciferase activity in lysates from THP-Mac expressing the wild-type Tat_SF2_ or Tat_SF2_1-72 and transfected with MAP2K3, MAP2K6-, or IRF7-luciferase reporter vectors in the presence or absence of WP631, a Sp1 inhibitor. “ + WP” indicates treatment with WP631 (0.3 ug/ml). **D**, **E**. Western Blot of Tat expressing cells at 8 and 16 h post infection, treated or not treated with Flavopiridol **(D)** or with WP631 **(E)**. Dotted lines indicate pairs of values with statistically significant differences.

We reported that Tat activation of ISG is mediated by Tat association with the promoters of MAP2K6 and MAP2K3, which activate p38MAPK, and of IRF7 [27]. This association leads to an increased accumulation of the corresponding RNAs and, in turn, to activation of the ISG [27]. Increased luciferase activity was observed when the luciferase gene was controlled by the MAP2K3, MAP2K6 or IRF7 promoter sequences and Tat was expressed in the APC [27]. In these constructs approximately 1000 bases of promoters sequences upstream the RNA start site were cloned at the 5′ of the luciferase gene [27]. Here we investigated to what degree the single-exon Tat_SF2_1-72 affected luciferase activity when its transcription is dependent from these promoters. Activation of the MAP2K3-Luc, MAP2K6-Luc, and IRF7-Luc promoter constructs was tested in THP-Mac cells using different Tat proteins. We found that luciferase activity was significantly higher in the presence of Tat_SF2_1-72 than of wild type Tat_SF2_ and that mutants in which the interaction with cellular transcription factors is compromised, such as Tat_SF2_C25,30,34S or Tat_SF2_K28,50A, could not stimulate luciferase activity at levels higher than those observed without any Tat protein (Figure 5A). These results are in agreement with the more robust activation of ISGs observed by this Tat mutant in iDC and MDM (Figures 2, 3C). We also found that there is variability in the level of stimulation of each of these promoters in cells from different donors (not shown). However, as all three gene products, MAP2K3, MAP2K6, and IRF7, can contribute to the activation of ISGs, these individual differences do not significantly affect the end result, when the induction of ISG is evaluated in different donor cells.

Tat interacts with cellular transcriptional regulators and it is possible that the increased transcriptional activity mediated by of MAP2K3, MAP2K6, and IRF7 promoters in the presence of Tat is a result of these interactions. To evaluate whether Tat-mediated gene expression modulation is affected when cellular transcription factors are inhibited, we used specific inhibitors to interfere with the function of P-TEFb, a factor required for cellular gene transcription, or Sp1, as all three promoters under investigation contain Sp1 binding sites. When THP-Mac transfected with the luciferase vectors were also exposed to Flavopiridol, inhibitor of CDK9 and therefore of P-TEFb [46], or WP631, inhibitor of Sp1 [47,48], the luciferase activities associated with both Tat_SF2_1-72 and wild type Tat_SF2_ were significantly reduced for all promoters (Figure 5B, C). Luciferase activity did not significantly diminish when compared between samples from untreated and treated cells that were not expressing Tat, although luciferase values were lower in the treated samples. These inhibitors did not affect Tat or β-actin levels of expression during the 16 h timeframe of the experiments (Figure 5D, E). We concluded that Tat stimulation of these promoters depends on Tat interaction with P-TEFb, which is compromised when the cysteine residues are mutated. Tat interaction with Sp1 also contributes to Tat-mediated gene modulation of these specific promoters. The one-exon version of Tat appears more efficient at this process than the wild type Tat, possibly by facilitating the activation of P-TEFb and Sp1.

### A HIV_SF2_ carrying a one-exon Tat stimulates the induction of IFN-associated genes more efficiently than wild type HIV

**Figure 6:**
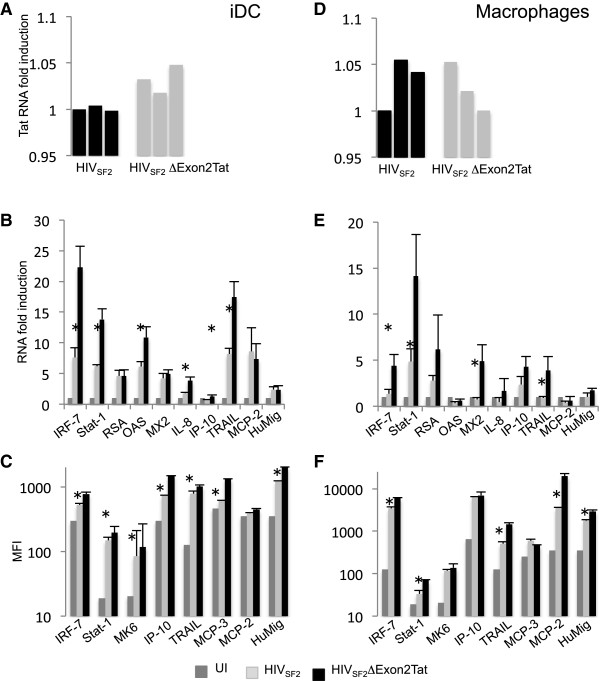
**A HIV virus lacking the second Tat exon induces a stronger innate response than a wild type virus. A**. Tat RNA levels in iDC 7 days after infection with HIV_SF2_ or HIV_SF2_ΔExon2Tat. **B**. ISG RNA levels in iDC infected for 7 days with HIV_SF2 or_ HIV_SF2_ ΔExon2Tat. Statistical significance was detected when comparing HIV_SF2_ and HIV_SF2_Δexon2Tat RNA values for IRF-7 (p = 0.03), Stat-1 (p = 0.006), OAS (p = 0.03,) IL-8 (p = 0.019), IP-10 (p = 0.05), TRAIL (p = 0.01), and for the ISG RNA values grouped together (p = 0.001) (*T*-test). **C**. Protein accumulation levels of ISG-related proteins in iDC infected with HIV_SF2_ or HIV_SF2_ΔExon2Tat on day 7 after infection, reported as MFI. Significant differences were found for IRF-7 (p = 0.01), Stat-1 (p = 0.007), MAP2K6 (p = 0.013), IP-10 (p = 0.01), TRAIL (p = 0.01), MCP-3 (p = 0.05), HuMig (p = 0.01), ISG protein values when grouped together (p = 0.0026). **D**. Tat RNA levels in MDM 7 days after infection with HIV_SF2_ or HIV_SF2_ΔExon2Tat. **E**. ISG RNA levels in MDM infected for 7 days with HIV_SF2_ or HIV_SF2_ ΔExon2Tat. Statistical significance was detected when comparing HIV_SF2_ and HIV_SF2_ΔExon2Tat RNA values for IRF-7 (p = 0.03), Stat-1 (p = 0.05), MX2 (p = 0.04) and (TRAIL p = 0.05), and for the cumulative levels of all the ISG (p = 0.0008). **F**. Protein accumulation levels of ISG-related proteins in MDM infected with HIV_SF2_ or HIV_SF2_ΔExon2Tat on day 7 after infection, reported as MFI. Significant differences were found for IRF-7 (p = 0.05), Stat-1 (p = 0.05), TRAIL (p = 0.01), MCP-2 (p = 0.0001), HuMig (p = 0.01), ISG protein values grouped together (p = 0.03). RNA fold induction represents the ratio between the RT-PCR result obtained for cells expressing Tat or Tat1-58 and that obtained for Ad-tTA infected cells. RNA amounts are normalized using 18S RNA levels and infection rates are normalized using Tat levels.

We investigated whether the more significant gene modulation observed with a single exon Tat compared to wild type Tat also occurred when a single exon Tat gene was part of the HIV genome (HIV_SF2_Δ Exon2Tat). When HIV_SF2_Δ Exon2Tat was used to infect iDC or MDM and levels of accumulated HIV transcripts in HIV_SF2_Δ Exon2Tat infected cells were compared to those obtained with HIV_SF2_ during a time course, there was initially a lower accumulation of HIV transcripts in the culture infected by the mutant. However by day 7-10 the amount of infected iDC or macrophage cells, estimated by staining with an anti-gp120 antibody, was similar for the two viruses and total Tat RNA accumulation varied little (Figure 6A, D). When ISG gene expression analysis was carried out by RT-qPCR, we found that expression of a number of them was significantly higher in iDC and MDM infected with HIV_SF2_Δ Exon2Tat compared to the same cells infected with wild type HIV_SF2_ (Figure 6B, E). When the cumulative induction of all the investigated genes by HIV_SF2_ was compared to that obtained with HIV_SF2_Δ Exon2Tat the difference was significant (p = 0.001 in iDC and p = 0.0008 in MDM). A higher level of induction of the corresponding gene products was also found when the intracellular protein accumulation was evaluated by ICS in HIV positive cells. The MFI was significantly higher in cells infected by HIV_SF2_Δ Exon2Tat (Figure 6C, p = 0.0026, 6F, p = 0.03). These data confirm the observations made with the expression of single exon Tat in the context of the full length HIV_SF2_: Tat second exon reduces the activation of cellular genes stimulated by Tat first exon.

## Discussion and conclusion

HIV infects a variety of cells that are critical components of the immune system and the outcome of the infection varies in different cell types. Cells like CD4+ T cells produce substantial amount of virus and undergo apoptosis, or die of cytopathic effect, or become latently infected; macrophages produce substantially less virus than T cells upon infection but are much more resistant to virus-mediated cell death; iDC produce even lower amounts of virus than macrophages and are also resistant to virus-mediated death. It is therefore logical to envision that these different cells respond differently to HIV or SIV infection and manage to do so by different adjustments of their gene expression program to the viral infection. Among the HIV proteins that could directly play a role in affecting cellular gene expression at the transcription levels there is Tat, a protein that regulates HIV and SIV gene expression and interacts with important components of the transcription machinery. We previously investigated Tat modulation of gene expression in iDC and T cells [23,29]. Here we compared Tat-mediated modulation observed in iDC to that of MDM and we addressed the question of whether specific domains of Tat are more critical than others in the host-pathogen interactions established by HIV in APCs and whether the induction of ISG is strictly mediated by Tat or could be mediated by the induction of IFNs. We found that there were no major qualitative differences of cellular gene modulation between the three 101 amino acid Tat proteins tested, both derived from clade B HIVs. Shorter Tat proteins had a more significant effect on cellular gene modulation than their full-length counterpart, supporting the role of Tat second exon in limiting Tat effects on cellular genes.

The mutants investigated here point at a mechanism of cellular gene transactivation similar but not identical to that used to increase efficiency of viral gene expression. The most important result is the evaluation of Luciferase activity stimulated by the promoters that are critical to the induction of ISG by Tat. We have shown that Tat associates with the promoters of MAP2K3, MAP2K6 and IRF7 and that this association is critical to the activation of ISG [27]. As only sequences upstream the RNA start sites were linked with the Luc gene, it is unlikely that an interaction with structural elements of the mRNA of these genes is critical to their transcriptional increase. This is also supported by the fact that TatA58T that is most efficient in transactivation of the HIV LTRs is not the most efficient modulator of cellular genes and it is actually less efficient that Tat_SF2_, Tat_HXB2_ or Tat_SF2_1-72, all of which transactivate the HIV LTR at lower levels than Tat_SF2_A58T (Figure 1). However, the fact that poor transactivators of the HIV LTR, such as Tat_SF2_C25,30,34S and Tat_SF2_K28A,K50A, also have a significantly lower effect on modulation of cellular genes and poorly stimulate luciferase activity controlled by MAP2K3, MAP2K6 and IRF7 promoters suggests that the interactions with p300 and P-TEFb are important for cellular gene modulation as they are for HIV efficient transcription. The results observed with the Tat_SF2_K28,50A mutant support the possibility that Tat acetylation and/or interaction with histone acetyl transferases are also necessary in order for Tat to modulate host cell mRNA transcription.

Tat-mediated modulation of cellular promoters appears to require interaction with P-TEFb, possibly favoring its switch from inactive to active and bypassing its regulation by cellular factors. Tat could facilitate promoter-proximal pause release, as it has been shown for Myc at many promoters [49,50] and for Tat at the cad promoter, where it can substitute Myc activation [49]. Each of the three promoters critical to Tat-mediated ISG activation contains Sp-1 binding consensus sequences. Therefore it is not surprising that the Sp1 inhibitor we tested reduced the luciferase activity they mediate. Tat promotes the phosphorylation of Sp1, which increases its binding to target sequences [19-21]. It is conceivable that Tat interaction with P-TEFb results in CDK9-mediated phosphorylation of Sp1, increasing its activity.

The luciferase activity experiments suggest a more important role of Tat in affecting the rate of RNA initiation rather than a change in elongation as it occurs at the TAR element of HIV transcripts. As all these genes are usually transcribed in the absence of Tat, it is not surprising that the stringent requirement of Tat to permit elongation of HIV transcripts does not apply to cellular RNAs. A detailed analysis of the complexes that include Tat at different promoters and lead to their increased transcription and what dictates the selectivity of different cellular promoters in different cell types will be the focus of future studies.

A single exon Tat modulated a larger number of genes and the magnitude of modulation was also more significant than that observed with a two exon protein. When coupled with the fact that the second exon is also necessary for efficient reverse transcription, these results provide an additional explanation for why a virus with a single exon Tat is quickly cleared in SIV infected animals [12]. The stronger innate response induced by a single exon Tat in infected APCs could facilitate stimulation of the adaptive response and virus clearance. Furthermore, among the ISG modulated by Tat are genes that significantly reduce retroviral replication such as PKR, OAS, and MX2, recently shown to have a profound effect on HIV infectivity [51-53]. The induction of ISG, which does not happen to the same extent in T cells [29], could explained the reduced viral replication that occurs in APC after infection with a wild type virus carrying a full length Tat, when reverse transcription is equally efficient in both T cells and antigen presenting cells. It may also explain why the more significant induction of these genes by a single-exon Tat and the consequent heighten ISG induction could further reduced retroviral replication in iDC and MDM. As the vast majority of infected T cells dies upon infection, a substantially reduced replication in APCs may be insufficient to sustain persistent infection. It is therefore to the virus advantage to modulate its replication in these cells in such a way that it is not too much, and therefore could lead to cell recognition and elimination by the anti-viral adaptive immunity or cell death, and not too little, with consequent progressive decline of viral persistence.

Our studies support the fact that Tat can mimic the role of IFN and can by itself activate pathways normally affected by viral pathogens signaling via Toll receptors. There are a number of reasons why this may be advantageous to the virus as, for instance, it may favor persistence of these infected cells by reducing the virus replication to a level that permits escape from the adaptive response. However this direct stimulation of innate immunity by Tat may also be responsible for the persistent immune activation and the more striking IFN signature observed in species that progress to AIDS [54-56]. It is unclear whether this happens in all species infected by lentiviruses or is more overt in species where the virus is pathogenic. When we analyzed the modulation of a limited subset of ISG genes in AIDS resistant species by HIV Tat or HIV and SIVmac, it appeared that their induction was not as clear-cut in AIDS-resistant species as it is in AIDS susceptible [57,58]. It is possible that coevolution of host and virus selected for viral or host proteins that avoid the effects that are most deleterious to the host. More extensive studies with matched viruses, Tat proteins, and primary cells from different primate species are necessary to reach a final verdict on this issue.

## Methods

### Cells and viruses

Peripheral blood mononuclear cells were isolated from blood by Ficoll-gradient centrifugation. Monocytes were isolated by negative selection with Monocyte Isolation Kit II (Miltenyi Biotec, Auburn, CA) according to the manufacturer’s instructions. The monocytes were differentiated into iDC by culturing them with human recombinant GM-CSF and IL-4 (R&D Systems, Minneapolis, MN) for six days [9] or into MDM, culturing them with human recombinant M-CSF (R&D Systems, Minneapolis, MN) for 7 days. HIV-1_SF2_ with the first exon of Tat truncated at position 59 and the second exon deleted was generated by three substitutions to the HIV-1_SF2_ sequence (accession number K02007): C6017T, C6026T, and C6035T. These substitutions replaced the amino acid glutamine with a stop codon at each position in the Tat sequence without affecting the amino acid sequence of Rev. HIV-1 infection was done with an amount of virus equivalent to 20 ng of p24 per 10^6^ iDC or MDM.

Recombinant adenoviruses Ad-tTA, Ad-Tat_SF2_, Ad-Tat_HXB2_, Ad-Tat_SF2_C25,30,34S, Ad-Tat_SF2_1-58, and Ad-Tat_SF2_1-72, Ad-Tat_SF2_A58T, Ad-Tat_SF2_E86-E92A, Ad-Tat_SF2_K28A,K50A, Ad-Tat_SF2_G48-R57A and their flagged version were constructed according to established protocols [59] and produced in 293 cells (ATCC^®^ CRL-1573™). The Tat coding region was cloned into the vector pAd-TRE-MCS1, which is a first generation serotype 5 adenovirus vector that has the genes E1 and E3 deleted. Tat is under a tetracycline inducible promoter in this vector and is expressed only in cells co-infected with Ad-tTA, which expresses the tetracycline responsive transactivator. 10^6^ iDC were infected with 5 plaque-forming units (PFU) per cell of both Ad-tTA and the Ad-Tat constructs. As a control iDCs were also treated with 100 U/ml of human IFN-α2a, IFN-β1a (PBL Biomedical Laboratories, Piscataway, NJ), and IFN-γ (R&D Systems, Minneapolis, MN). The K562 cell line (ATCC^®^ CCL-243™), which lacks the entire locus for Type I interferons [60], was used to elucidate the role of Type I IFNs play in the observed gene modulation [61-63]. The U5A cell line was a gift of Dr. George Stark (Cleveland Clinic).

### RNA isolation and quantitative RT-PCR

The growth media of cells and the cells were collected 0 h, 5 h, 10 h, 20 h, and 30 h post infection. The cells were collected in Trizol^®^ (Invitrogen, Carlsbad, CA) to isolate total RNA according to the manufacturer’s instructions. The RNA was treated with 4 U of DNase I (Ambion, Austin, TX) and purified with Trizol^®^ LS. 200 ng of RNA was used as a template in reverse transcription with iScript cDNA Synthesis Kit (Bio-Rad, Hercules, CA). cDNA was amplified using primers specific for Tat, the selected subset of ISG, IFN-α, IFN-β, and IFN-γ. For amplification of IFN-α cDNA we used primers that anneal to regions conserved in the subtypes. Real-time PCR was performed with the iTaq SYBR Green Supermix With ROX kit (Bio-Rad, Hercules, CA) in the ABI Prism 7000 Sequence Detection System (Applied Biosystems, Foster City, CA). Glyceraldehyde 3-phosphate dehydrogenase (GAPDH), β-actin, or 18S RNA levels were used to normalize the amount of RNA in samples. The levels of Tat mRNA were used to normalize the levels of infection among different cultures. To measure the number of IFN-β mRNA copies in infected iDC, we compared the relative RNA amount measured by quantitative RT-PCR to IFN-β mRNA standards. To generate the standards, we cloned the IFN-β gene under a T7 promoter and made mRNA with mMessage Machine kit with poly A tailing kit (Ambion, Austin, TX) according to the manufacturer’s instructions.

### Gene expression analysis

The RNA from iDC and MDM infected with the Ad-Tats was analyzed with the Human Genome U133 Plus 2.0 Array and RNA from iDC infected with HIV-1 with the Human Genome U133 Array (Affymetrix, Santa Clara, CA). Values in the raw data below 100 were floored to 100. Expression levels were compared to RNA from uninfected cells and cells infected with Ad-tTA alone as a control. Gene up-regulation was examined only for transcripts from Ad-Tat infected cells that were present according to the detection call and down-regulation only for genes that were present in the uninfected and Ad-tTA-infected controls.

### ELISA

Cytokine levels were measured in cell culture growth medium by ELISA, performed according to the manufacturer’s instructions. We used the following ELISA kits: human CXCL10/IP-10 and CXCL9/MIG DuoSet^®^ ELISA Development System (R&D Systems, Minneapolis, MN), Human IFN Beta ELISA Kit (PBL Biomedical Laboratories, Piscataway, NJ) and Human Interferon-β ELISA Kit (TFB, Inc., Tokyo, Japan). The p24 concentration of the amplified HIV-1 virus was measured with HIV-1 p24 ELISA kit (PerkinElmer™ Life Sciences, Inc., Boston, MA). LTR transactivation in HeLa-CD4-LTR-β-gal cells was measured with β-Galactosidase Enzyme Assay System with Reporter Lysis Buffer (Promega, Madison, WI).

### Luciferase assay

For luciferase assays, cells were transfected with MAP2K6- (-991 to -1 nucleotides from MAP2K6 start site), MAP2K3- (-1013 to -1), IRF7- (-1018 to -1) luciferase vectors (SwitchGear Genomics, Carlsbad, CA, cat. # S710112, S718628, S721774) and then infected with Ad-tTA or Ad-Tat_SF2_, Ad-Tat_SF2_1-72, Ad-Tat_SF2_1-58, or Ad-Tat_SF2_K28,50A and Ad-tTA. Cell lysates were assayed for firefly and *Renilla* luciferase activities (Promega, Madison). For the CDK9 inhibition, cells were treated with Flavopiridol (Alvocidib) at 70 nM (AdooQ.com Biosciences, Irvine, CA). In the case of the Sp1 inhibition, cells were treated with WP631 dihydrochloride at 0.3ug/ml (Santa Cruz Biotechnology, Dallas, TX).

### Western blotting

SDS-PAGE gel electrophoresis of the cell lysates from THP1-Mac infected with the Ad-Tat_SF2_ and Ad-Tat_SF2_ 1-72 was carried out in the presence or absence of Flavopiridol or WP631 according to published procedures [64]. An anti-FLAG antibody was used for Tat detection and anti-B-actin antibody as control (Santa Cruz Biotechnology, Dallas, TX).

### Analysis of ISG-related protein expression by intracellular cytokine staining (ICS)

After 7 days of infection with HIV_SF2_ or HIV_SF2_Δ2exonTat the cultures were treated with Brefeldin A (BD Biosciences) the 16 h before staining and FACS. Anti HIV gp120 Strain IIIB FITC (US Biological) was used to stain infected cells and set the gate for the Env + cells only. After permeabilization with Perm solution (BD bioscience) cells were ICS stained with the followings antibodies: efluor660-MCP-2, PE-HuMig, PE-MCP-3 (eBioscience), Alexa647-IRF-7, Pacific blue-Stat-1, PE-IP-10, PE-TRAIL (BD biosciences) and uncogugated-MAP2K6 (Abcam) was used with a PECy7-conjugated secondary antibody. Flow cytometric analysis to evaluate MFI was performed using FACS Canto (BD Biosciences) and FlowJo v9.1 (TreeStar, Ashland, OR).

### Statistical analysis

Calculations and statistical analyses were performed using GraphPad Prism version 3 software. Between-group comparisons were carried out by two-tailed, *t* test or Mann-Whitney test. Within group comparisons were done by one-way ANOVA followed by Bonferroni post-hoc test. Results of statistical analyses were considered significant if they produced *p* values ≤ 0.05.

## Competing interests

The authors declare that they have no competing interests.

## Authors’ contributions

SK generated the adenoviral vectors and the single exon HIV, carried out the gene expression analysis of the mutants and IFN in iDCs, and drafted the initial manuscript and some of the figures. MDEPMV carried out comparative evaluation of Tat_SF2_ and Tat_Bal_ and the corresponding single exon protein in MDM, luciferase assays and evaluated the role of inhibition of CDK9 and Sp1 during the same experiments, and prepared some of the figures. NK carried out the comparative analysis of Tat-mediated gene modulation in iDC and MDM, the evaluation of Tat in K562 and prepared some of the corresponding figures. MM carried out evaluation of HIV and its corresponding mutant virus with a one-exon Tat, and ELISA assays for the evaluation of different cytokine production in supernatants of cells expressing Tat alleles and mutants. AA conceived of the study, secured funding for it, and participated in its oversight, coordination, trouble shooting and in the editing of the manuscript and figures. All authors read and approved the final manuscript.
